# Protecting Parents, Idealizing the Past, Blaming Friends: Life Stories of Men Imprisoned for Violence

**DOI:** 10.1177/0306624X231198802

**Published:** 2023-09-22

**Authors:** Carolina Agoff, Matias Bruno, Sveinung Sandberg

**Affiliations:** 1 National Autonomous University of Mexico (UNAM), Ciudad de Mexico, Mexico; 2 Escuela IDAES-UNSAM, Buenos Aires, Argentina; 3 University of Oslo, Norway

**Keywords:** narrative analysis, life-stories, violence, psychosocial criminology, narrative criminology, Latin America

## Abstract

Life-stories emerge from a wide variety of facts and events in individual lives and weave a selected few of these together to make meaning in the present. They are crucial for constructing identity and influence action by establishing worldviews and a persona that narrators will seek to confirm. In this study we describe three main themes in the life-stories of six incarcerated men in Argentina: a) Protecting family, especially parents; b) reconstructing an ideal past, and contrasting it with a more cynical present; and c) blaming criminal neighborhoods, friends, and girlfriends for their crimes. We discuss how these themes are intertwined, what function they fulfill, and the identities and masculinities they produce. Combining research on life-stories with narrative and psychosocial criminology the analysis reveals how life-stories of incarcerated men can be seen as attempts at countering stigma and defending a self that is under attack. The life-stories portray a believable, ‘good’, and multifaceted image of the self, but most importantly, create coherence and unity in otherwise chaotic lives.

## Introduction

Antonio (34) is serving an extended sentence for various homicides, most of which were of police officers. He had lived a violent life, and this shaped his life-story. At the age of 13, Antonio was already long into his criminal career and had gotten hold of a gun. His father, who himself had just been released from prison, punished him for carrying the weapon. The beating was so bad that Antonio was hospitalized, and the injuries so serious that he still carries them with him. In revenge, he attacked his father and tried to kill him: “I shot him as soon as I got out of the hospital,” Antonio explained: “That’s when I got my personality. I gained a reputation in the neighborhood. Nowadays a kid is respected when he kills someone.” What followed in Antonio’s life seems cinematic, but is nevertheless true: years later, the police killed his father, leaving his body in full view of his family. From then on, Antonio went out to “hunt” police officers to avenge his father’s death. For every cop he killed, he tattooed a tomb on his back. At the time of the interview, he had seven.

A narrative is often defined as a sequence of at least two events, where one causes the other (Polletta et al., 2011). The first event in Antonio’s life-story is how his father punished him, the second is Antonio’s revenge, and the third, how he avenged his father’s murder. It is a form of “quest narrative,” where suffering is met head on and used to motivate a mission, often for a greater goal (Frank, 2013). The story also merges different parts of his life into one, gives coherence to the sequence of events, and presents a strong, violent, and heroic masculinity. Much like other life-stories, Antonio’s narrative thus provides his life “with unity and purpose by constructing internalized and evolving narratives of the self” (McAdams, 2001, p. 100). The way Antonio had imprinted the story onto his body revealed its importance for him. The story’s plot is a version of Freudian proto-fantasy of an archaic legacy (Loustau, 1976); the beating from the father leading to an attempted father-murder, which is turned on its head when the father is killed, and Antonio avenges his death. The life story also functions and does important *narrative work*, as described by Frank (2010).

When interviewed in prison in Argentina, Antonio defended his father and emphasized that it was not his father who had led him to commit crimes and homicide. Although he acknowledged that his father was in prison “for 15 years for attempted murder, robbery, shooting, injuries,” he explained that he now realized “why my father beat me up.” Antonio excused him by saying that it was “because he didn't want the same thing to happen to me.” Who was then to blame? “In the neighborhood, there were kids with cars, who had all the girls, everyone waved at them.” He continued: “You want to do the same (. . .) It was a bad example! It gave me prestige, it gave me status in the neighborhood: to have a gun, a brand-new car, and four girls around, money.” Antonio distinguished between the past and present when describing the criminal environment of his neighborhood: “Before, we kids were all friends in the neighborhood, we smoked a joint. Nowadays they’re all at each other’s throats. Today respect is lost,” he claimed, which echoed the opinion expressed in many other interviews also conducted for this study.

The aim of this study is to identify some main themes in life-stories told by men convicted of violent offences—and to discuss their function. One important theme is the construction of a violent street or protest masculinity that serves as a source of both self-respect and respect from others. This has been well covered in extant literature (e.g., Connell, 2005; Messerschmidt, 1993; Mullins, 2006). We therefore focus instead on themes that were common, but that have been less explored in criminology, such as: protecting family and reconstructing an ideal past. These were related to finding culturally acceptable explanations for crimes, which is the third theme we explore. The aim is to move beyond a singular emphasis on neutralization (Maruna & Copes, 2005), and violent masculinities (e.g. Andersson, 2008; Lauger, 2014), to explore some less studied—but important—parts of violent life-stories.

## Life-Stories: Narrative and Psychosocial Criminology

Everyday storytellers seldom have explicit strategies or aims for their narration. They may mimic and get carried away by stories they have heard, and often attempt to be coherent and resonate with their audience. Storytelling is chaotic, dynamic, situational, and performative (Bal, 2009), and only rarely is it conscious or calculated. Nevertheless, stories still perform particular tasks for storytellers, which may explain why particular episodes or themes are chosen, the particular plot or direction, and why they are told in a particular way.

McAdams (2012, p. 15) famously outlined an approach to life-narratives in which “people construct and internalize stories to make sense of their lives” and where “these autobiographical stories have enough psychological meaning and staying power to be told to others as narrative accounts.” The perspective emphasize that life-stories can be analyzed for “content themes, structural properties, functional attributes,” and used to understand “psychological, social and cultural meanings.” It is highlighted that life-stories create coherence and integrate lives. McAdams (2006) also mentions “the redemptive self,” a particular identity which is important for marginalized lives. Redemption can entail re-signifying negative experiences (e.g., violence, prison, poverty) in order to create positive subjective outcomes (learning something or changing positively).

Chase (2005) describes how stories are both conditioned by social context and impact social interaction. Life-stories are accomplishments or forms of action that express agency at the same time as they are fundamentally shaped by socioeconomic and discursive structures. To reflect the agency of storytelling, Frank (2010) describes what these stories do as narrative work. Although this work is rarely conscious, or a strategy on the part of the storyteller, it is still work with traceably real effects on people’s lives. Similarly, Gubrium and Holstein (2009, p. 39) use the metaphor of work to capture storytelling. They describe how it highlights that the storyteller “actively orients to a task,” also emphasizing that it is “not done self-consciously.” Following these narrative scholars, narrative work may then be better understood akin to what Bourdieu (1990) describes as habitus, or deeply internalized structures of behavior and thought. Fleetwood (2016, p. 173) for example, links habitus to narrative theory, by highlighting that stories, as other competencies, are “embodied, learned and generative.”

We combine this emphasis on life-stories and their narrative work with narrative and psychosocial criminology (Binik & Verde, 2025; Verde, 2020; Verde & Knechtlin, 2019). Narrative criminology is an emerging framework that often uses the life stories of people convicted of crimes as data. The approach emphasizes that these stories are complex and plurivocal (Sandberg, 2010), but still essential to understanding the lives, offences, and futures of people who have been in contact with the criminal justice system (Fleetwood et al., 2019; Presser & Sandberg, 2015). The psychodynamic psychosocial framework (Brunner et al., 2012; Pohl, 2010; Winter, 2018) builds on this, and emphasizes the unconscious forces of the human mind upon which male subjectivity is founded and that underlie and motivate life-stories. Such a perspective is also helpful in understanding aspects of crime that are unreducible to group identities (Gadd & Jefferson, 2007). Maruna and Copes (2005), for example, highlight the importance of studying the unconscious to understand neutralization, an approach with many parallels to the well-established study of defense mechanisms in psychoanalytic theory (S. Freud, 1930, 1933; A. Freud, 1936).

Narrative criminology understands stories as “vehicles for self-understanding and as such an instigator to action” (Presser, 2009, p. 191), while psychosocial criminology has emphasized that analysis “needs to be based on the notion of a psycho-social subject whose meanings and actions are produced in relationships—relationships which are not fully accessible to intention and conscious knowledge” (Hollway, 2001, p. 21). Despite this and other differences, Verde and Knechtlin (2019) point to many important similarities between narrative and psychosocial perspectives. These include an emphasis on the multifaceted self, the co-construction of stories, and how people strive to be seen as coherent and “good.” Importantly, however, the self that is created in life-stories is “an imaginary project: always unfinished, fragmented, with relevant alien parts, and looking for an imaginary unity” (Verde, 2020, p. 54). As exemplified by Antonio’s story, concepts such as defense mechanisms and narrative creativity illustrate how the selection and organization of life-stories can be seen as (mostly) unconscious narrative work to create coherence in chaotic lives.

In this study, we explore some common themes in the life stories of six men incarcerated for violence in Argentina. The analysis emphasizes how these themes connect the present and the past and the role they play for the storyteller—or what narrative work they perform. Finally, we show how they contribute to particular subjectivities or selves. Following Binik and Verde (2025, pp. 291–316), we focus on narratives that respond to stigmas and trace these back to past and traumatic experiences. In this way we show how these life-stories serve to construct intrapersonal coherence and defend a challenged self.

## Methods

The data in this study are drawn from six interviews with men convicted of serious violent offenses. The interviewees were selected from two different studies of people in prison in Argentina: one from 2017 and the second in 2022. All interviews lasted between 120 and 180 minutes. In the first study, participants were interviewed once, while in the second study, participants were interviewed 3 times (for more details, see Author’s reference removed). Interviews were conducted in closed spaces, with only the interviewer and the interviewee present. Participants were allowed to speak relatively freely about their lives and only guided indirectly by an interview guide. This study also draws on insights from other interviews done with violent men (and women) in these two projects. We chose these six men to enable a more in-depth examination and to provide more biographical detail, which are important for both narrative and psychosocial criminological analyses.

In both studies, the interviews were organized as life-story interviews covering participants’ lives from childhood to the present (Atkinson, 1998). The advantages of life-story interviews are that they provide access to “personal conceptions of the past and all its stages,” and that they are “readily interpretable” (Tagg 1985, p. 163). Kohli (1981) further points out that the description of events in temporal order aids the interviews because interviewees typically expect some kind of organization to a conversation. For this study, life-stories are the topic of our inquiry itself, and hence especially important. The life-story methodology is helpful because it “documents personal experiences and transformations over time” and arguably allows “revealing what other methods hide or obscure” (Messerschmidt, 2000, pp. 16–17). We rely on insights from narrative criminology, when finding that incarcerated individuals (as most other people) have a broad repertoire of stories that play out differently, depending on the positionality of researchers and storytelling contexts (Brookman, 2015; Sandberg et al., 2015). However, most people still have a rather limited narrative repertoire (Sandberg, 2010) and lengthy and sometimes repeated interviews will tend to reflect some of these general themes (Damsa & Ugelvik, 2017). Below we explore a few important themes in the life-stories of the six incarcerated men in this study.

## Three Forms of Narrative Work

Antonio (34), Cristian (29), Luciano (41), Roberto (38), Luis (42), and Marcelo (38) belong to a generation that grew up in a social and economic context of great change. The “neoliberal” Argentina of the 1990s had left, among other marks, a significant increase in crime and insecurity, widespread drug use in vulnerable sectors, and an opening in the market for goods and services that exacerbated economic and social inequalities. The six men came from lower and lower-middle class families and had grown up in urban contexts of relative deprivation. They did not lack necessities, such as food, housing, and education, and thus early crime was mainly a strategy to obtain secondary consumer goods. They attended school like most children but dropped out as their criminal involvement increased. Between the ages of 13 and 15, all those interviewed had already left school and committed crimes such as robbery or even homicide. By this age, three of them had left the home in which they had grown up.

Within such a context of relative poverty, crime and imprisonment, storytelling may seem irrelevant or of less importance. At the same time, their dramatic experiences invite narrative understanding, both for themselves and others. Below we lay out how they exempt their parents (especially mothers) from any responsibility for their crimes, while blaming the social environment (shantytowns) and “criminal” friends or girlfriends for their criminal activity. Their life-stories shift between attempts at neutralizing or justifying, and embracing a violent self. In what follows we detail these themes and discuss the narrative work (Frank, 2010) they do in life-stories and for storytellers.

## Protecting Family

Against the backdrop of suburban neighborhoods, characterized by precarious housing (shantytowns), and other social housing, Cristian, Antonio, and Luis emphasized that their families were hard-working people with a strong work culture, and that their relatives had tried hard to shield them from crime. Their fathers, uncles, and grandfathers were factory workers, nurses, and bakers. They were part of a large working-class population, “at that time they were fashionable,” said Antonio. Shots were sometimes heard in the neighborhood: “But thank God” Cristian said, “my dad was a worker and he kept us . . . or wanted to keep us out of it.”

Cristian characterized his neighborhood as “all dirt, lots of soccer. You could see everything. You could hear shots . . .” He was the oldest son of a large family from the countryside. His grandmother took care of him. Cristian describes her as “a country woman” with good values related to hard work and sacrifice. Bakers by trade, the whole family worked. His mother was a housewife: “the old belief, that’s how we were raised.” His parents got along well. They were used to another culture, working in the fields, educating the children, and beating them if they misbehaved: “I stole candy when I went shopping and told my dad. He beat me a lot. He gave us everything we wanted. He took me to clubs.” For Cristian the beating he received was a legitimate part of raising children and the story was woven into a larger portrayal of a father who gave his children “everything,” as a traditional, family man, who instilled values such as honesty and sports, and gave the best of himself.

Luis recalls his parents’ effort to take him out of the shantytown, and send him to secondary school outside the neighborhood “as a way of leaving the slum, of not belonging to the slum,” and added that the experience of many fellow prisoners were similar: “Our parents always had that hope that their son will be a doctor, something, some profession, and there was always this question of hiding the address where we come from.” Marcelo says that it was not until he became an adult that he understood the role played by his parents: “Now that I’m grown up, you see, now I sort of understand it a little more. They tried to instill in us, that we have to work, that we have to study, about those things, you see?” Roberto similarly came from a family with many economic difficulties and at the age of 12 he was drawn towards the street. He similarly excused his family from the criminal path he had taken:

I remember that my mother and my stepfather never encouraged me to have this life for which I am here today, no. My uncles came, we had family dinners, we went to the house of my other uncles, we had no problem with the law.

Unlike the other interviewees, Luciano came from a middle-class family and emphasized that it was not only those who come from “the slums” who steal. Everywhere there are “working people” and “those who steal,” he assured. Although his background was different, he also pardoned his family for his crimes:

I was getting used to a way of life. My mum used to say: “Easy come, easy go.” She never took money from me, my mom. She never liked it, but she never locked me up either. She did what she could, my sisters turned out well.

Luciano stated explicitly that he had the opportunity to have another life, “Nobody forced me to do what I did!” he said with certainty. Such an emphasis on being in charge of one’s own life can be seen as the official discourse of prison, and prisoners use this to “pay lip service” to trends in correctional programming (McKendy, 2006). In an increasingly individualist and neoliberal society, however, similar ideas of individual responsibility are far-reaching (Wetherell & Potter, 1992). The emphasis on agency is also a main component of masculinity (e.g. Connell, 2005) meaning that anyone who does not take such responsibility might be accused of being “less of a man.”

Järvinen (2001, 2003) argues that many accounts might best be understood if they are related to real or imaged accusations. Many of the participants in this study did not locate the origin of their criminal career in a bad relationship with their parents, family violence, or neglect. Instead, they protected their family from such imagined (but likely) accusations by explicitly exempting them from any responsibility for their crimes and misbehavior. The narrative work of “protecting and defending mummy” (Verde & Knechtlin, 2019) might be a psychological mechanism to alleviate any guilt they feel towards their parents. Roberto, for example, had great remorse regarding his mother: “when the moment came, when she passed away, I remember the moments when my mother came (to prison) and I saw her sad face.” From a psychodynamic point of view, such conflicts between their criminal lifestyle and the internalization of mainstream norms and ideals produce permanent feelings of guilt (Freud, 1930). If such tensions are not resolved, some propose it can lead to mental illnesses or violence (Brunner et al., 2012). Exempting their parents from blame through narrative work in life stories could be one way to resolve this tension.

## Reconstructing an Ideal Past

Descriptions of good parents were tightly linked to stories of a good childhood. The two themes came together as a striking feature of these life-stories: a nostalgia for the past and a narratively constructed opposition between an idealized childhood and a present characterized by cynicism and problems. Oftentimes, the life of the previous generations was seen as a model of honesty and hard work, as opposed to the world of criminal actions they were now immersed in. There was also a common story about the difference between criminal environments “back in the days” and “now.” Cristian said:

I made myself respected, because I was quiet, and people knew that I stole. And because of my father, who was a straight man, and everybody respected him. The kids (respected me) for doing the things I did, and the grown-ups, because I had good manners.

Christian said that in his teen years and early youth, the codes that governed the neighborhood were “to challenge the other kids, who steals more, who steals less, it was another mentality.” The honesty of his parents was still intertwined with the “honesty” or “innocence” of criminal life in that time. They seemed to go together in a past that was presented as being fundamentally different from the present in terms of moral values and qualities.

Cristian’s story shows a certain longing for the criminal life in those years, characterized by less violence: “I would mug people in cars, whatever the person had, he would let it go. It is not like now. Before, it was quieter.” Luciano also recalled with nostalgia the codes that governed those times:

There was respect for the one who stole the most, not the most violent. The group respected you, but also other people (even those who didn’t steal). The one who stole the most was the smartest and they flattered you. And if you steal and buy a motorbike, they see you and start to respect you.

As part of this story the present was described as being more violent and cynical. According to many of the participants, today there is no friendship between street kids, and respect is instead earned by killing someone bigger and better known. Luciano continued:

At that time there were other codes. I stole without weapons and so did many others. There were few who stole with guns. Nowadays they kill you for nothing. At that time there were codes that were respected (. . .) Before there was evil too, but you made yourself respected by fighting and that was it. You could also be respected for being generous, a good friend.

Although Antonio described his criminal past in less “innocent” ways, with violence and murders an integrated part of his story, he still pointed out that there were important differences:

Yes, there is companionship. A companion is faithful. A comrade kills for his comrade. He kills and dies for his comrade. That is the most loyalty there is. At that time, I can tell you that it did exist. But today, it exists with an interest. Let’s go to gossip and then boom, everyone goes on with his life. And then you go to jail and your partner fucks your wife. Where is the friendship? Where is the loyalty that existed before?

The idealization of the past can also be observed through the way that participants view petty crime and compare it to previous times with more planned, smarter, and bigger crime. Marcelo said: “Most of the things that happen *now* are not good crimes. I think young people are involved in other things.” He added “I get angry when they say someone stole a cell phone” and described them as petty thieves stealing from honest people, “the prison is full of them, of minor crimes.” In an association of ideas, Marcelo stated with great passion:

We must walk with good irons (guns), the good irons are going to give us the exit, we are not going to go to jail, for me it was always that. I always rejected the creeps you saw, who go and steal from a working kid who is standing at the bus stop. I always repudiate them, I reject them, I fuck them up with irons if I see them. I make a mess of everything, I don't like it, maybe because my old man was a worker too, right?

In these performances of violent masculinity, well known in this literature (e.g., Mullins, 2006), he also includes a respect for workers (like his father), a disdain for petty thieves, and pride in the ownership of powerful weapons associated with street culture and crime.

The idealization of the past, and especially childhood, is widespread in many segments of the population (Grundetjern & Tchoula, 2022; Hyman, 2014). It may be a reminder of lost innocence, a response to unwanted change, or a storytelling technique to narrate tragic drama (Frye, 1957). Brow (1990, pp. 2–3) notes that almost everywhere “the sense of belonging together is nourished by being cultivated in the fertile soil of the past.” So, these stories might also be an attempt to connect with a broader community. From a psychoanalytical perspective, however, such idealization is the result of an intrapsychic and unconscious defense mechanism that serves to ward off aggressive impulses against feelings of guilt and anxiety. Idealization of the past is particularly striking in this study. Given the context in which they grew up, it is sometimes difficult to imagine the harmony they describe. Over and above, or perhaps in addition to, descriptions of actual changes, it is interesting to see how reconstructing an ideal past was combined with “protecting” close family members while blaming peers and girlfriends, as these coincide in time. Idealization of the past make these elements of their life stories come together more coherently.

## Blaming the Criminal Environment, Peers, and Girlfriends

Protecting parents from blame and idealizing the past is important, as it entails taking responsibility for one’s own actions. However, most people in the criminal justice system will also, and often simultaneously, try to find external reasons for their situation. Such “excuses” make it easier to live with the harm they have caused, and arguably, also to move on (Maruna & Copes, 2005). Close family members, especially mothers, seem to be “off limits,” but there were other factors and people that appeared easier to blame. In such stories, there is an element of the classic “not my fault” neutralization technique (Sykes & Matza, 1957), but we argue that a psychosocial criminological approach also adds additional insights to such accounts.

The men suggested that there were limits to what they could become due to the poverty, classism, and racism that locked them into a category of “black slum dwellers.” They felt that the stigma afforded them only two options: to play soccer or steal. Their exclusion was seen as leading them to a criminal career, often seen as shaped by circumstances which they saw as beyond their control. Antonio, for example, pointed out that: “I lived stigmatized, I was discriminated against and that's where the construction begins, you get kicked out of school, and they make you see that you are a slum dweller and that gets into you.”

The combination of poverty and discrimination is a well-known driver of street culture and associated crime (Anderson, 1999; Bourgois, 2003). The criminal environment is also prevalent in the explanations for crime embedded in the life-stories of the men in this study who were convicted of violent offenses. In addition to pointing to some obvious and important circumstances that led them into a life of crime, these accounts also help to relieve them of individual responsibility for the harm they have caused. Cristian talked about how he first smoked marihuana out of curiosity at the age of 14. In his life-story, he places this event as the start of something he described as a life of crime, drug use and violence. He emphasized the bad influence of a cousin and a group of friends:

We were in this stupidity with my older cousin who was in prison. I met him when he got out, at an aunt’s house. My father told me not to go there. “I don’t want to see you there,” he said. And that’s where I went, and I tried marihuana (. . .) I started smoking every day. I already had my little group to smoke with. That’s when I got out of control. I had a chance, but I went off the rails.

Cristian explained how he spiraled downwards from then on, and began stealing to be able to use. The themes of protecting family (in this case the father) and taking responsibility for his own choices (“I had a chance”) are present, but he combines these explicitly in his story with blame for others. The cousin is not deserving of the same “protection” as other family members, and is instead put in the same category as the friends who influenced him badly. Luciano, similarly, started smoking marihuana at 13, repeated the year and dropped out of school. In the neighborhood he smoked with his age group and saw older kids stealing: “Seeing the environment, seeing the older kids stealing stereos, and I don’t know if it was because of repetition, contagion, or imitation, I started stealing stereos, bicycles. I started to like the street.”

Marcelo left home at the age of 15 to live in a shanty town and attributed his starting to commit crimes to this social environment, together with a desire for material goods: “Yes, I can say by living there I began to have more of a relationship with, let’s say, crime. In the rush to move forward. I wanted a car.” Having friends with longer criminal records was also described as an important reason for criminal careers. Luis said:

A guy came out of being in jail, a colleague, and he tells me, I heard that you were pinching banks, well we’re going to rob one (. . .) I knew it was wrong because a lot of police, guns, I come to jail, 10, 12, 15 years old, noooo. I’m showing off, it was just to show the guy, no more. Look at what happened!

Girlfriends or other girls were also sometimes described as a negative influence. An unplanned teenage pregnancy triggered a conflict between Cristian and his father:

At the age of 16 I fell in love, and she got pregnant. My father was against it, and I told him “I’m going to take charge” and I left home. And I started stealing every day because I was living on the street and with another younger boy. A couple of months. Then I went back to my grandmother’s house. I gave money to my girlfriend.

The dream of a family life first led him to leave his family, then to stealing, and later, after her infidelity, to psychological and drug-related problems: “She was unfaithful with my friends. I was crying, I didn’t know what to do. I had taken cocaine and tried to hang myself.” This episode further increased his drug use and lead to a period of intense criminal activity. In explaining how they had come to crime, several men talked about getting into financial trouble, wanting to impress, being deceived by, or lured into crime by existing or potential girlfriends.

Blaming socio-economic marginalization, discrimination, a criminal environment, deviant peers and even girlfriends for having ended up in a criminal career and subsequently in prison was common. In a realist criminological framework, the emphasis would be on if and how these stories reflect the main reasons for their criminal engagements. In a narrative and psychosocial perspective however, regardless of their reliability, the same stories can be seen as defense mechanisms providing welcome neutralizations (Sykes & Matza, 1957) or accounts (Scott & Lyman, 1968) of the harm they had committed and their current incarceration. Attributing guilt to a criminal milieu, peers and even girlfriends seemed to be more acceptable than blaming parents. In psychosocial terms, there might be a relation between feelings of guilt, defense mechanisms (projection of guilt on peers), and guilt relief by blaming others rather than parents. This complexity of narratives and emotions includes social conditions, the phenomena, and the inner contradictions of the subject (Brunner et al., 2012). These three themes or forms of narrative work we have described are connected in that parents and childhood is associated with the idealized past while criminal environment, friends and girlfriends are associated with the much-criticized present.

## Discussion

It is difficult to generalize from six participants, but many of the themes in these interviews also resonate with the larger samples from which these interviews were drawn. Despite important exceptions, there was a strong tendency to downplay the role of parents (especially mothers) and instead to blame the environment, peers, and girlfriends for the crimes committed, as well as a tendency to idealize the past. In criminological literature, narrative attempts at avoiding blame are usually described as neutralization techniques (Sykes & Matza, 1957) or accounts (Scott & Lyman, 1968). To our knowledge however, shielding significant others from blame and idealizing the past have not been addressed in depth in the criminological literature (but see Grundetjern & Tchoula 2022; Verde & Knechtlin, 2019). Arguably, the narrative work of these stories is crucial for marginalized people, and may be better understood when coupled with narrative psychology and psychosocial criminology.

McAdams (2006) emphasizes that one of the most important roles of life stories is to create identity coherence. Following a long tradition of studies of stories in criminology, the life stories of imprisoned people also serve to justify criminal behavior, both to a societal audience and to themselves (Maruna & Copes, 2005). According to psychosocial theory, both these purposes are pre-reflexive or unconscious and have intrapsychic and intersubjective functions (Hollway, 2001). Inspired by psychosocial criminology (Binik & Verde, 2025; Verde, 2020; Verde & Knechtlin, 2019), we interpret these findings through an explanatory model of psychic unity and integrity. The major and recurring themes across the six interviews are the maintenance of a coherent identity when confronted with the painful experience of the prison, and the associated ego failure. Being stigmatized and in prison requires compensatory defense mechanisms to stabilize the psychic equilibrium.

Grundetjern and Tchoula (2022) describes nostalgia as a cultural symptom of collective anxiety associated with loss of control. Similarly, imprisonment is a milestone that often establishes a before and an after point in life stories. It can provoke projections of a non-existent past that represents an ideal with which to confront a frustrating present. This is a defense mechanism that, in the form of a dichotomy, confronts past and present, family, and extra-familial relationships (friends and women) to keep an identity afloat that helps inmates cope with the prison environment. Idealization of the past can be a defensive response to the insufficiency and vulnerability of the present, and the consequent need for compensation and realization of desire on the level of the phantasmatic imaginary. To preserve ego-strength and inner unity, coherence between the ideal past and the frustrating present is crucial. Alternatively, the result can be narcissistic mortifications, as failures and frustrations can only be explained by individual deficiency (Roock, 2013). Notably, feelings of guilt appear to be mainly associated with parents, without any mention of remorse for their victims. The effort to exonerate parents from all responsibility for their crimes and to present them as honest people, reveals the guilt of not having submitted to the family authority structure and instead having satisfied their own narcissistic desires. At the same time, the criminal social environment functions as a “projective identification,” that serves both as the motivation and as the excuse for criminal behavior (Poppi & Verde, 2021, p. 16).

Life stories balance the need to simplify a life, with the need to still convey some of its complexity. As explained by McAdams (2006, p. 118): “Life is messier and more complex than the stories we tell about it. Yet the stories need to convey some of that complexity if they are to be viewed, by the self and by others, as credible and life-affirming.” Arguably, the three themes we have identified contribute to creating coherence and to simplify, while at the same time, convey some of the complexity of life. They do the narrative work of both psychological defense mechanisms (S. Freud, 1930, 1933; A. Freud, 1936) and neutralization techniques (Sykes & Matza, 1957) by addressing the primary guilt felt towards parents, and project responsibility for the damage onto others. When the past is idealized, it merges the three stories, moving from a childhood with good parents to an adolescence with problematic friends, and thereby creates narrative order and identity coherence.

We have emphasized three themes. As mentioned more briefly however, these themes are usually intertwined or even overshadowed by a presentation of a violent masculinity in these life-stories. It seems that the ideal of a heroic, defensive, and ready-to-fight-masculinity is present when feelings of powerlessness and rejections appear, or when the men feel attacked and pushed towards feelings of undeniable dependence (Winter, 2018, p. 84). When protest (Connell, 1991) or street masculinity (Mullins, 2006) is added into the mix of these life stories, as seen for example in the quotes from Antonio and Marcelo, this nuances a self-presentation as “good” (in more conventional terms), contributing to a more complex self, and making their life stories more believable, in line with McAdams’s (2006) argument (see also Hermans, 1996; Rosenwald, 1992). Together, these life stories thus convey both coherence and complexity, which serve as a reminder of the similarities between narrative criminology (Fleetwood et al., 2019) and psychosocial criminology (Gadd & Jefferson, 2007). As pointed out by Verde and Knechtlin (2019) this includes a multifaceted image of the self and a drive towards being seen as coherent and “good.”

## Conclusion

Antonio’s story, which we began with, is exceptional and extreme in many ways—but when explored in detail, it contains many familiar themes that we recognize from years of research with marginalized populations. Most importantly, this includes the protection of parents, an idealization of the past, blaming environment, friends and girlfriends for criminal pathways, and the construction of a violent masculinity. Since violent masculinity is often emphasized in criminological research, especially in research on men in prison (e.g. Ugelvik, 2014), we have emphasized the three first dimensions of these life-stories. Together, these themes create a narrative structure that builds biographical coherence and attempts to counter societal stigmatization. Despite the deviations conveyed in these stories, there are many similarities to the way that more conventional lives are told. This makes sense both within a perspective of neutralization theory (lawbreakers share values with mainstream society) and within a psychosocial perspective. McAdams (2006) argues (p. 121) a life story “needs to be told from a recognizable moral perspective.” In this way these stories can also serve as a bridge between lives considered deviant, and more conventional lives.

The three themes we have identified illustrate how life stories are both a window into individual lives and experiences, and illustrative of more shared storytelling structures. Although culture and social context is crucial for storytelling, we will argue that these stories may possibly reveal some more universal characteristics of narrative and psychosocial structures. Such patterns of unconscious motivations and affects may thus be relative independent of social and cultural specificities. Idealizing the past for example, seems to be a common human tendency that reflects a longing to turn away from the dependencies and limitations of everyday reality. However, one important difference is that while a lot of people are critical of their parents, it seems that these marginalized and imprisoned men—who probably could blame their parents for a lot—seemed to have a more urgent need to protect them. While being an early observation at this point, it might be worth exploring more in future narrative and psychosocial criminological studies.

## References

[bibr1-0306624X231198802] Andersson K. (2008). Constructing young masculinity: A case study of heroic discourse on violence. Discourse & Society, 19(2), 139–161.

[bibr2-0306624X231198802] Anderson E. (1999) Code of the street: Decency, violence and the moral life of the inner city. W.W. Norton.

[bibr3-0306624X231198802] Atkinson R. (1998). The life story interview. SAGE Publications. 10.4135/9781412986205

[bibr4-0306624X231198802] Bal M. (2009). Narratology. Introduction to the theory of narrative. University of Toronto Press.

[bibr5-0306624X231198802] Binik O. Verde A. (2025). Female murderers and stigma: Coping with the “Bad Woman” label. International Journal of Offender Therapy and Comparative Criminology, 69(4), 291–316. 10.1177/0306624X221124842 36222586

[bibr6-0306624X231198802] Bourdieu P. (1990). The logic of practice. Polity Press.

[bibr7-0306624X231198802] Bourgois P. (2003). In search of respect: Selling crack in El Barrio. Cambridge University Press.

[bibr8-0306624X231198802] Brow J. (1990). Notes on community, hegemony, and the uses of the past. Anthropological Quarterly, 63(1), 1–6.

[bibr9-0306624X231198802] Brookman F. (2015). The shifting narratives of violent offenders. In Presser L. Sandberg S. (Eds.), Narrative criminology: Understanding stories of crime (pp. 207–234). New York University Press.

[bibr10-0306624X231198802] Brunner M. Burgermeister N. Lohl J. Schwietring M. Winter S. (2012). Psychoanalytische Sozialpsychologie im deutschsprachigen Raum. Geschichte, Themen, Perspektiven. Freie Assoziation, 15(3–4), 15–78.

[bibr11-0306624X231198802] Chase S. (2005). Narrative inquiry: Multiple lenses, approaches, voices. In Denzin N. Lincoln Y. (Eds.), The sage handbook of qualitative research (pp. 651–679). SAGE Publications.

[bibr12-0306624X231198802] Connell R. W. (1991). Live fast and die young: The construction of masculinity among young working-class men on the margin of the labour market. The Australian and New Zealand Journal of Sociology, 27(2), 141–171.

[bibr13-0306624X231198802] Connell R. W. (2005). Masculinities. Routledge.

[bibr14-0306624X231198802] Damsa D. Ugelvik T. (2017). A difference that makes a difference? Reflexivity and researcher effects in an all-foreign prison. International Journal of Qualitative Methods, 16(1), 1609406917713132.

[bibr15-0306624X231198802] Fleetwood J. (2016). Narrative habitus: Thinking through structure/agency in the narratives of offenders. Crime, Media, Culture, 12(2), 173–192.

[bibr16-0306624X231198802] Fleetwood J. Presser L. Sandberg S. Ugelvik T. (Eds.). (2019). The emerald handbook of narrative criminology. Emerald Group Publishing.

[bibr17-0306624X231198802] Frank A. W. (2010). Letting stories breathe: A socio-narratology. University of Chicago Press.

[bibr18-0306624X231198802] Frank A. W. (2013). The wounded storyteller: Body, illness, and ethics. University of Chicago Press.

[bibr19-0306624X231198802] Freud A. (1936). Das Ich und die Abwehrmechanism (The ego and the mechanisms of defence). Karnac Books.

[bibr20-0306624X231198802] Freud S. (1930). Das Unbehagen in der Kultur (Civilization and its discontents). Gesammelte Werke.

[bibr21-0306624X231198802] Freud S. (1933). Neue Folge der Vorlesungen zur Einführung in die Psychoanalyse (New introductory lectures on psychoanalysis). Internationaler Psychoanalytischer Verlag.

[bibr22-0306624X231198802] Frye N. H. (1957). Anatomy of criticism. Princeton.

[bibr23-0306624X231198802] Gadd D. Jefferson T. (2007). Psychosocial criminology. SAGE.

[bibr24-0306624X231198802] Grundetjern H. Tchoula W. (2022) Nostalgia and rumors in the rural methamphetamine market. American Journal of Cultural Sociology, 10, 492–515.

[bibr25-0306624X231198802] Gubrium J. F. Holstein J. A. (2009). Analyzing narrative reality. SAGE.

[bibr26-0306624X231198802] Hermans H. J. M. (1996). Voicing the self: From information processing to dialogical interchange. Psychological Bulletin, 119(1), 31–50.

[bibr27-0306624X231198802] Hollway W. (2001) The psycho-social subject in ‘evidence based practice’. Journal of Social Work Practice: Psychotherapeutic Approaches in Health, Welfare and the Community, 15(1), 9–22.

[bibr28-0306624X231198802] Hyman L. (2014). Happiness and memory: Some sociological reflections. Sociological Research Online, 19(2), 1–9.

[bibr29-0306624X231198802] Järvinen M. (2001). Accounting for trouble: Identity negotiations in qualitative interviews with alcoholics. Symbolic Interaction, 24(3), 263–284.

[bibr30-0306624X231198802] Järvinen M. (2003). Negotiating stragerhood: Interviews with homeless immigrants in copenhagen. Acta Sociologica, 46(3), 215–230.

[bibr31-0306624X231198802] Kohli M. (1981). Biography: Account, text, method. In: Bertaux D. (Ed.) Biography and society (pp. 61–75). Sage.

[bibr32-0306624X231198802] Lauger T. R. (2014). Violent stories: Personal narratives, street socialization, and the negotiation of street culture among street-oriented youth. Criminal Justice Review, 39(2), 182–200.

[bibr33-0306624X231198802] Loustau R. J. (1976). Protofantasy. Acta Psiquiatrica Psicologica de America Latina, 22(2), 121–132. 983741

[bibr34-0306624X231198802] Maruna S. Copes H. (2005). What have we learned from five decades of neutralization research? Crime and Justice, 32, 221–320.

[bibr35-0306624X231198802] McAdams D. P. (2001). The psychology of life stories. Review of General Psychology, 5(2), 100–122.

[bibr36-0306624X231198802] McAdams D. P. (2006). The problem of narrative coherence. Journal of Constructivist Psychology, 19(2), 109–125.

[bibr37-0306624X231198802] McAdams D. P. (2012). Exploring psychological themes through life-narrative accounts. In: Holstein J. A. Gubrium J. F. (Eds.), Varieties of narrative analysis (pp. 15–32). SAGE.

[bibr38-0306624X231198802] McKendy J. P. (2006). “‘I’m very careful about that’: Narrative and agency of men in prison.” Discourse and Society, 17(4), 473–502.

[bibr39-0306624X231198802] Messerschmidt J. W. (1993). Masculinities and crime: Critique and reconceptualization of theory. Rowman & Littlefield Publishers.

[bibr40-0306624X231198802] Messerschmidt J. W. (2000). Nine lives: Adolescent masculinities, the body, and violence. Westview.

[bibr41-0306624X231198802] Mullins C. W. (2006). Holding your square: Masculinities, streetlife and violence. Willan Publishing.

[bibr42-0306624X231198802] Pohl R. (2010). Männer – das benachteiligte Geschlecht? Weiblichkeitsabwehr und Antifeminismus im Diskurs über die Krise der Männlichkeit. In Bereswill M. Neuber A. (Eds.), In der Krise? Männlichkeiten im 21. Jahrhundert. Westfälisches Dampfboot.

[bibr43-0306624X231198802] Polletta F. Chen P. C. B. Gardner B. G. Motes A. (2011). The sociology of storytelling. Annual Review of Sociology, 37(1), 109–130.

[bibr44-0306624X231198802] Poppi F. I. M. Verde A. (2021). Odi et amo: Discursive strategies and ambiguity in the narratives of violence. European Journal of Criminology, 18(6), 918–939.

[bibr45-0306624X231198802] Presser L. (2009). The narratives of offenders. Theoretical Criminology, 13(2), 177–200.

[bibr46-0306624X231198802] Presser L. Sandberg S. (Eds.). (2015). Narrative criminology: Understanding stories of crime. NYU Press.

[bibr47-0306624X231198802] Roock M. (2013). Männlichkeit in der Krise? Zur Bedeutung von männlicher Geschlechtsidentität und Weiblichkeitsabwehr in sich transformierenden Arbeitsverhältnissen. Psychologie und Gesellschaftskritik, 36/37(4/1), 151–174.

[bibr48-0306624X231198802] Rosenwald G. C. (1992). Conclusion: Reflections on narrative self-understanding. In: Rosenwald G. C. Ochberg R. L. (Eds.), Storied lives: The cultural politics of self-understanding (pp. 265–289). Yale University Press.

[bibr49-0306624X231198802] Sandberg S. (2010). What can “lies” tell us about life. Notes towards a framework of narrative criminology. Journal of Criminal Justice Education, 21(4), 447–465.

[bibr50-0306624X231198802] Sandberg S. Tutenges S. Copes H. (2015). Stories of violence: A narrative criminological study of ambiguity. British Journal of Criminology, 55(6), 1168–1186.

[bibr51-0306624X231198802] Scott M. B. Lyman S. M. (1968). Accounts. American Sociological Review, 33(4), 46–62. 5644339

[bibr52-0306624X231198802] Sykes G. M. Matza D. (1957). Techniques of neutralization: A theory of delinquency. American Sociological Review, 22(6), 664–670.

[bibr53-0306624X231198802] Tagg S. (1985) Life story interviews and their interpretation. In Brenner M. Brown J. Canter D. (Eds.), The research interview: Uses and approaches (pp. 163–196). Academic Press.

[bibr54-0306624X231198802] Ugelvik T. (2014). Paternal pains of imprisonment: Incarcerated fathers, ethnic minority masculinity and resistance narratives. Punishment & Society, 16(2), 152–168.

[bibr55-0306624X231198802] Verde A. (2020). Whose narratives? Tijdschrift over Cultuur & Criminaliteit, 10(3), 35–58.

[bibr56-0306624X231198802] Verde A. Knechtlin N. (2019). Protecting and defending mummy’: Narrative criminology and psychosocial criminology. The Emerald Handbook of Narrative Criminology. Emerald Publishing Limited.

[bibr57-0306624X231198802] Wetherell M. Potter J. (1992). Mapping the language of racism: Discourse and the legitimation of exploitation. Harvester Wheatsheaf.

[bibr58-0306624X231198802] Winter S. (2018). Verdraengen, um zu herrschen. Eine psychoanalitisch-sozialpsychologische Betrachtung. Mittelweg, 36(4), 68–86.

